# In-Silico Integration Approach to Identify a Key miRNA Regulating a Gene Network in Aggressive Prostate Cancer

**DOI:** 10.3390/ijms19030910

**Published:** 2018-03-19

**Authors:** Claudia Cava, Gloria Bertoli, Antonio Colaprico, Gianluca Bontempi, Giancarlo Mauri, Isabella Castiglioni

**Affiliations:** 1 Institute of Molecular Bioimaging and Physiology, National Research Council, 20090 Segrate (Mi), Italy; claudia.cava@ibfm.cnr.it (C.C.); gloria.bertoli@ibfm.cnr.it (G.B.); 2 Interuniversity Institute of Bioinformatics in Brussels (IB)^2^, 1050 Brussels, Belgium; axc1833@med.miami.edu (A.C.); gbonte@ulb.ac.be (G.B.); 3 Machine Learning Group (MLG), Department d'Informatique, Universite libre de Bruxelles (ULB), 1050 Brussels, Belgium; 4 Department of Informatics, Systems and Communication, University of Milan-Bicocca, 20125 Milan, Italy; mauri@disco.unimib.it; 5 SYSBIO Centre of Systems Biology (SYSBIO), 20126 Milan, Italy

**Keywords:** prostate cancer, microRNA/miRNA, copy number alterations, co-expressed genes

## Abstract

Like other cancer diseases, prostate cancer (PC) is caused by the accumulation of genetic alterations in the cells that drives malignant growth. These alterations are revealed by gene profiling and copy number alteration (CNA) analysis. Moreover, recent evidence suggests that also microRNAs have an important role in PC development. Despite efforts to profile PC, the alterations (gene, CNA, and miRNA) and biological processes that correlate with disease development and progression remain partially elusive. Many gene signatures proposed as diagnostic or prognostic tools in cancer poorly overlap. The identification of co-expressed genes, that are functionally related, can identify a core network of genes associated with PC with a better reproducibility. By combining different approaches, including the integration of mRNA expression profiles, CNAs, and miRNA expression levels, we identified a gene signature of four genes overlapping with other published gene signatures and able to distinguish, in silico, high Gleason-scored PC from normal human tissue, which was further enriched to 19 genes by gene co-expression analysis. From the analysis of miRNAs possibly regulating this network, we found that *hsa-miR-153* was highly connected to the genes in the network. Our results identify a four-gene signature with diagnostic and prognostic value in PC and suggest an interesting gene network that could play a key regulatory role in PC development and progression. Furthermore, *hsa-miR-153*, controlling this network, could be a potential biomarker for theranostics in high Gleason-scored PC.

## 1. Introduction

Prostate cancer (PC) is a leading cause of cancer mortality in men and the most commonly diagnosed male malignancy [1]. When diagnosed at an early stage of the disease, PC is potentially curable by radical prostatectomy, which involves the removal of the prostate gland, and/or by radiotherapy.

Currently, the only circulating protein biomarker routinely used for the early diagnosis of PC is the prostate-specific antigen (PSA). The expression level of this serum biomarker measured at diagnosis has been proven to correlate with disease aggressiveness [2].

However, PSA has some restrictions. Several nonmalignant processes, including benign prostatic hyperplasia (BPH) and prostatitis, which occur in many men as they age, frequently lead to serum PSA increment, limiting the specificity of PSA for cancer detection [3]. Moreover, the optimal threshold of PSA expression level per biopsy is not clear [4]. Furthermore, PSA screening can lead to over-diagnosis and overtreatment of indolent prostate cancers [5,6,7].

Currently, some biomarkers found through a gene profiling approach have been proposed in clinical oncology. Many studies demonstrated that they could be used with both diagnostic and prognostic purposes.

A promising gene biomarker of PC is the prostate cancer gene 3 (*PCA3*) [8]. The automated PCA3 urinary assay, named ProgensaTM PCA3, is already clinically available [9,10]. *PCA3* has been found highly overexpressed in malignant PC tissue compared with PC benign and normal tissues. However, the detection of *PCA3* expression ignores the heterogeneity of cancer development and may only notice a proportion of PC cases [11,12].

Looking for new potential PC biomarkers, three of the most studied modifications in PC cells are found in gene expression, copy numbers, and microRNAs (miRNAs) expression. Aberrant expression of genes, due to copy number alterations (CNAs) and miRNA expression alterations, has frequently been reported in cancer [13]. CNAs contribute to cancer by altering the function of genes or pathways that are crucial for tumorigenesis, metastasis, and resistance to therapies. miRNAs act in different biological functions including development, proliferation, differentiation, and cell death [14,15]. Moreover, miRNAs have the advantage to be measurable in body fluids, such as blood or urine. With respect to gene profiling or CNA, this feature makes miRNAs suitable and non-invasive potential biomarkers for cancer diagnosis, prognosis, and treatment [16].

Being miRNAs stably expressed in circulating biofluids, such as plasma, serum, or urine, their analysis overcomes the stability challenges presented by total mRNA. Indeed, to isolate not-degraded mRNA, clinical tissue samples need to be immediately snap frozen after surgery, to maintain mRNA stability. On the contrary, miRNAs have been demonstrated to be stable in different conditions of temperature and pH [17,18]. Of course, to have reliable results, the preparation, handling, and storage of biofluid samples should be standardized to avoid confounding variables influencing the results. Several problems are linked to the different methods used for miRNA extraction and amplification (i.e., microarray analysis versus RT-PCR), as well as to the miRNAs chosen as internal references. For PC, *miR-16* and *miR-141* have been proposed as reliable references for the analysis of PC circulating miRNAs [19]. Regarding specifically *miR-153*, this miRNA has been already isolated in circulating biofluids (i.e., plasma and whole blood) in pathological conditions, although not related to PC [20,21,22].

Regarding PC, several groups focused on gene expression analysis [23,24,25]. True et al. [23] identified a signature of 86 genes to discriminate between low-grade and high-grade PC. Cuzick et al. [24] used the expression of 31 genes altered in PC to define a new score based on a pre-defined cell cycle progression and assessed the prognostic value of this score. The score is a robust index of the proliferative activity in the tumour and could have a central role in determining suitable treatments for PC patients. Penney et al. [25] built a 157-gene signature to improve outcome predictions and reduce overtreatment of PC.

Other groups focused on copy number alterations [26,27,28,29,30,31]. Beroukhim et al. [26] reported a comparison of CNAs among 26 different cancer diseases and determined that PC has more CNAs than most of the other considered cancer types. Frequent deletions were found on chromosome 6q, 8p, 10q, and 13q involving *NKX3-1*, *PTEN*, *BRCA2*, and *RB1* genes [27]. Previous studies reported that expression of oncogenes might be increased as a result of DNA amplification, and expression of tumor suppressor genes might be inactivated by physical deletions of DNA sequences [26,27,28,29,30,31].

Epigenetic alterations, such as miRNA level changes, are frequent in PC [4]. Several miRNAs have been identified to be differentially expressed between normal and PC [32,33,34,35]. Schaefer et al. [33] identified 15 miRNAs differentially expressed between PC and adjacent normal tissue. This signature was able to classify the two tissue types with an accuracy of 82%.

The target genes of prognostic miRNAs show altered expression profiles similar to those of the genes used for PC prognosis [36]. In particular, previous studies, which analysed PC-altered gene pathways, found that some prognostic miRNAs have their target genes enriched (a group of highly interconnected genes) in prognostic modules [36]. Each of these miRNAs might act as a master regulator of a gene pathway, i.e., regulating the behaviour of the whole module through the targeting of one or more single gene components [36,37].

The combination of gene expression, CNAs, and miRNA expression was approached in few studies and only in some cancer diseases, but not in PC, leading to interesting results in terms of tumour classification [38,39,40,41,42,43,44]. A combinatorial approach in PC is the one of Taylor et al. [45], which used the information from gene profiling, CNA, and miRNA in order to investigate the most altered gene pathways in PC.

Considering that there are many other factors that affect the gene expression (e.g., epigenetic regulation, repression from transcriptional factor, DNA methylation), in this study, we focused on the assumption that tumour heterogeneity is not only due to a simple accumulation of genetic alterations but can be the result of the combined effect of genetic and epigenetic alterations. Several studies support the validity of this theory in cancer. Published studies [46,47,48] suggested that the loss of a single functional allele is insufficient to perturb cellular functions and that the second allele can be silenced by epigenetic modifications.

Furthermore, if more factors interfere in the expression of a key gene, it is more likely that this gene will undergo a change. Key genes involved in cancer development are more likely subjected to several possible modifications (i.e., CNA, miRNA, …). Since different factors are able to modify them, these genes can be easily deregulated.

In this work, we investigated, in silico, the properties of mRNAs and miRNAs within a network of co-expressed genes, deregulated as an effect of aggressive PC. The gene network was selected by an integrative approach combining mRNA expression profiles, CNAs, and miRNA expression levels. miRNAs controlling this network could be potential biomarkers for PC theranostic applications.

## 2. Results

### 2.1. Gene Expression, miRNA, and CNA Analyses

Quantile analysis identified 15398 mRNAs and 760 miRNAs. The gene expression analysis of aggressive PC versus normal samples (NS) identified 3069 deregulated genes. Among these, 1735 were found to be downregulated and 1334 were found to be upregulated in PC patients. In this phase we obtained the expression levels of the up- or downregulated mRNAs as identified from gene expression analysis.

miRNA analysis of aggressive PC versus NS identified 239 deregulated miRNAs: 177 upregulated miRNAs (up-miRNAs) and 62 downregulated miRNAs (down-miRNAs). We identified mRNA targets of each deregulated miRNA. We identified 12,318 unique putative mRNA targets of 177 up-miRNAs and 10,087 unique putative mRNA targets of 62 down-miRNAs.

CNA analysis revealed 457 deleted genes and 168 amplified genes.

### 2.2. Combination of Gene Expression and CNA

In this phase, we determined the expression levels of the upregulated genes presenting amplifications and of the downregulated gene characterized by deletions, as identified from the combined analysis of gene expression and genome CNA.

Up- and downregulated genes with CNAs were selected allowing the identification of 38 deregulated genes. Specifically: 14 upregulated genes with copy number gains and 24 downregulated genes with copy number losses were found in PC patients.

Table 1 shows these genes with their alterations and positions in the genome.

### 2.3. Combination of Gene Expression or CNA and miRNAs

Amplified- and deleted genes that are target of deregulated miRNAs were selected allowing the identification of 178 deregulated genes. Specifically: 33 amplified genes, target of downregulated miRNAs, and 145 deleted genes, target of upregulated miRNAs, were found in aggressive PC patients.

Up- and downregulated genes, target of deregulated miRNAs, were selected allowing the identification of 1739 deregulated genes. Specifically: 554 upregulated genes, target of downregulated miRNAs and 1185 downregulated genes, target of upregulated miRNAs, were found in aggressive PC patients.

One miRNA (hsa-miR-876), which was downregulated with a deletion in DNA codifying for the pri- or pre-miRNA, was found from the combination of deregulated miRNA and their CNAs.

### 2.4. Combination of Gene Expression, CNA, and miRNA

In this phase, we identified the expression levels of upregulated and amplified genes that are target of down-miRNAs, and the expression levels of downregulated and deleted genes that are target of up-miRNAs.

We found, by the combination of gene expression, CNAs, and miRNAs, 21 genes: 3 upregulated and amplified genes that are target of down-miRNAs, and 18 downregulated and deleted genes that are target of up-miRNAs.

Table 2 shows these genes with their alterations and miRNA target.

**Figure 1:**
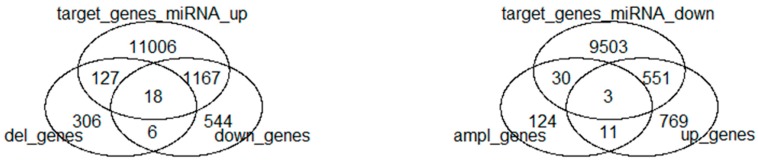
Venn diagram for the integrative approaches.

Figure 1 shows the Venn diagram of the combined approaches.

### 2.5. Prostate Cancer Signatures

From Pubmed Search, we obtained four previously published gene signatures associated with our 21 genes [49,50,51,52]. Based on the comparison with Mashima et al. [49], Rizzi et al. [50], Duhagon et al. [51], and Özdemir et al. [52], a downsized gene signature was found from our 21-gene signature, including only genes in common with the above considered gene signatures. Table 3 shows the published considered gene signatures.

The four-gene-based gene signature consisted of Tribbles pseudokinase 1 (*TRIB1*), Clusterin (*CLU*), Kruppel-like Factor 5 (*KLF5*), and Ephrin receptor A3 (*EPHA3*) genes. *TRIB1* was included in the Mashima et al. [49] signature. Using a functional genomic approach applied to the 3D spheroid cell culture model, the *TRIB1* gene was identified as an essential factor for PC cell growth and survival. The *CLU* gene was included in the Rizzi et al. [50] signature, consisting in an eight-gene signature detected by real-time quantitative PCR from 41 PC patients. These genes distinguish PC from benign tissue. *KLF5* was included in the Duhagon et al. [51] signature composed of 66 genes that characterize LNCaP cell line and PC patients. *EPHA3* was included in the three gene signatures of Özdemir et al. [52] which, associating the molecular signature of the stroma response in PC-induced osteoblastic bone metastasis, highlights the expansion of hematopoietic and prostate epithelial stem cell niches.

### 2.6. Co-Expressed Network

**Figure 2:**
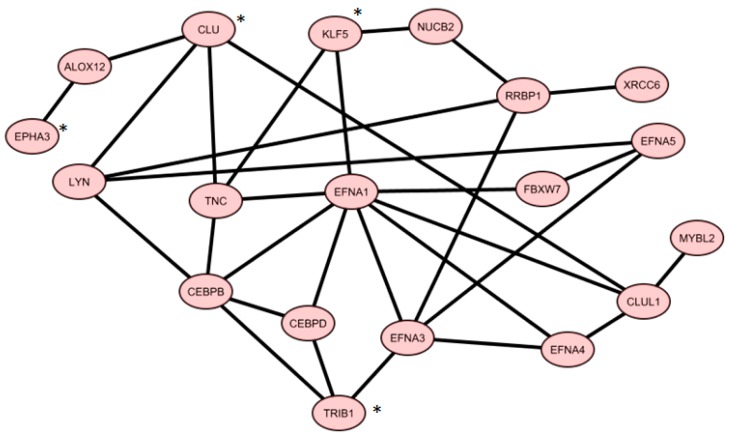
Co-expression gene network of core genes from four gene signatures overlapping with published gene signatures (* four gene signatures).

From Gene Mania analysis using *TRIB1*, *CLU*, *KLF5*, and *EPHA3*, we achieved a co-expression network, containing 19 genes shown in Figure 2.

Table 4 shows how this network was constructed, according to the Gene Mania database.

In total, we identified 386 miRNAs with target genes belonging to the co-expressed gene list. In this way, we generated a miRNA list.

Then, we focused on miRNAs with a significant number of target genes belonging to the same co-expressed gene list.

**Figure 3:**
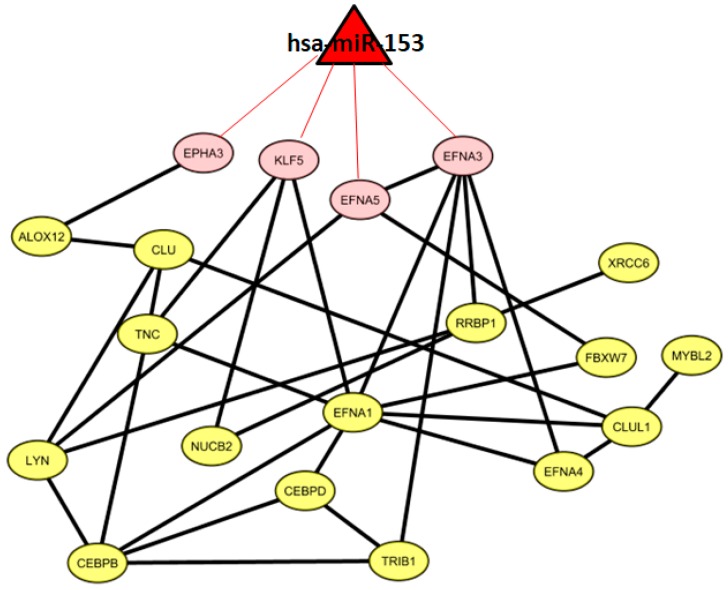
Gene co-expression network and putative miRNA-regulated targets.

We found one miRNA (*hsa-miR-153*) that could control a sub-pathway of the co-expression network. In particular, *hsa-miR-153* could regulate four genes, namely, *EPHA3*, *KLF5*, *EFNA5*, and *EFNA3*. Figure 3 shows 19 co-expressed genes and the miRNA regulator.

### 2.7. Classification of Normal and Aggressive Prostate Cancer Samples

**Figure 4:**
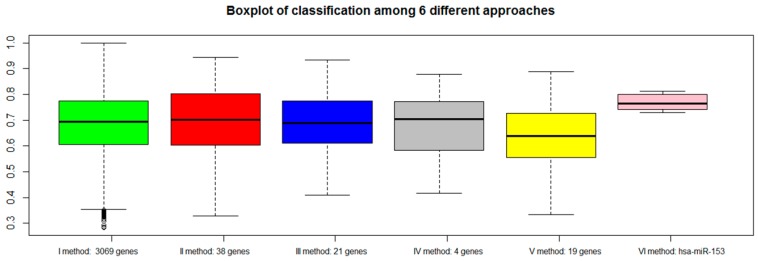
Area Under Curve (AUC) values of six different approaches: green bar (I method with 3069 genes), red bar (II method with 38 genes), blue bar (III method with 21 genes), gray bar (IV method with 4 genes), yellow bar (V method with 19 genes), and pink bar (VI method with *hsa-miR-153*).

For each approach (I: gene expression, II: combination of gene expression and genome CNA, III: combination of gene expression, genome CNA, and miRNA analysis, IV: genes overlapping with other gene signatures, V: co-expressed gene list, VI: co-rank miRNA list), the Area Under Curve AUC results of normal versus aggressive PC classification are presented in Figure 4. For the VI approach (miRNA signature), we used *hsa-miR-153*.

Although the combination strategy allowed to reduce the number of genes from 3069 to 21 (from I to III), all genes signatures derived by the five approaches (I, II, III, IV, V, VI) achieved good performance, but better results were found for method VI (*hsa-miR-153*). Approach IV, which selected a lower number of genes overlapping with published gene signatures (four genes), performed well (third quartile around AUC 0.80).

**Figure 5:**
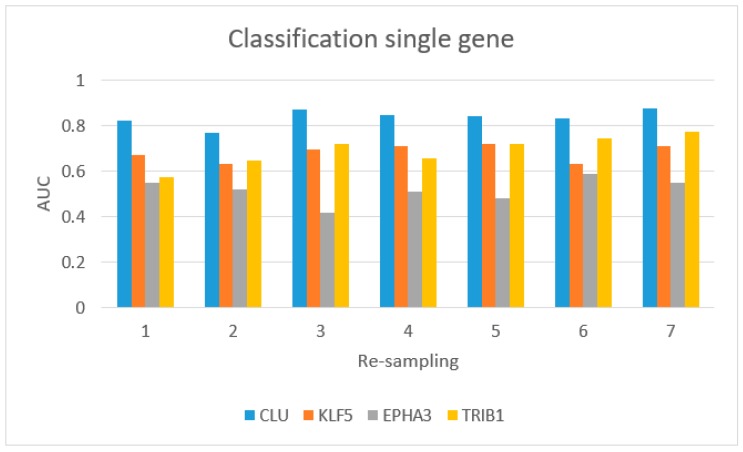
Classification for single gene from the IV approach (four gene signatures): blue bar (*CLU* gene), red bar (*KLF5*), gray bar (*EPHA3*), and yellow bar (*TRIB1*).

Figure 5 shows the AUC values for single-gene classification, using *CLU*, *KLF5*, *EPHA3*, and *TRIB1*. *CLU* achieved the best performance.

Additional file 1 shows the performance of classification of the four-gene-based signature in patients with Gleason score 6 versus controls and Gleason score >8 versus controls. We achieved the best classification using the four-gene-based signature to distinguish patients with Gleason score >8 from controls.

**Figure 6:**
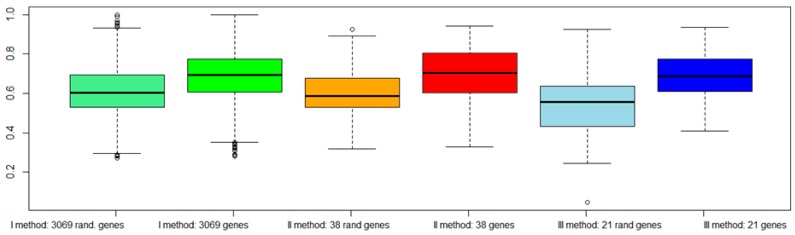
AUC values among three different approaches with dataset TCGA, considering also a subset of random genes. The light green box represents AUC with 3069 random genes, and the dark green box AUC with 3069 genes according to our approach. The orange box represents AUC with 38 random genes, and the red box AUC with 38 genes according to our approach. The light blue box represents AUC with 21 random genes, and the dark blue box AUC with 21 genes according to our approach.

**Figure 7:**
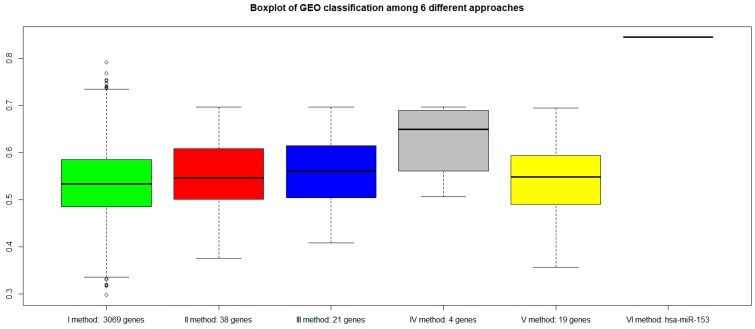
AUC values of six different approaches with a validation dataset GEO (GSE79021 for gene expression, and GSE21036 for miRNA): green bar (I method with 3069 genes), red bar (II method with 38 genes), blue bar (III method with 21 genes), gray bar (IV method with 4 genes), yellow bar (V method with 19 genes), and pink bar (VI method with *hsa-miR-153*).

To evaluate the validity of the proposed approaches: (a) We classified the same dataset TCGA considering a subset of genes randomly chosen among the dataset (Figure 6); (b) We classified an independent dataset from GEO considering the gene signatures selected by our procedures (Figure 7).

Figure 6 shows a worsening of the classification performance when random genes were chosen with respect to genes selected by our procedures.

Figure 7 shows that all genes signature maintained similar performances. However, the VI method (with *hsa-miR-153*) showed a better AUC performance.

## 3. Discussion

In this work, we investigated the properties of genes and miRNAs in PC, selected with the use of different combination approaches, including the integration of mRNA expression profiles, CNAs, and miRNA expression levels in the co-expressed network. Since PC of low Gleason score (3+3) does not metastasize, it is never lethal, thus the clinical conundrum in caring for men diagnosed with PC is to identify aggressive diseases with lethal potential; we thus focused the work on PC with GS 7 or higher.

To better clarify the genes and miRNAs which are altered in aggressive PC versus NS and the role of these genes and miRNAs in PC development, we initially found the mRNAs altered in aggressive PC versus NS (I approach) with CNA (II approach), reducing the number of interesting genes from 3069 to 38 (Table 1).

Among those genes, we observed that there were several genes already described having a role in PC, such as *PVT1*, whose increased expression is associated to PC [53] or *CLU*, and *GSTM1*, which has been already proposed as a PC biomarker [54,55]. Moreover, we noticed that, among the region highly affected by genome amplification and deletion, chromosome 1 and 8 were frequently present.

Previous studies have also shown that chromosome 8 alterations, as 8p21-22 and gain of 8q24, are commonly reported in PC. FISH analysis suggested that alterations of chromosome 8 are statistically significantly associated with PC stage III [56].

Chromosome 1 was demonstrated to contain PC susceptibility genes [57]. In particular, three PC susceptibility genes have been reported to be linked to different regions on chromosome 1: *HPC1* at 1q24-25, *PCAP* at 1q42-43, and *CAPB* at 1p36.

The 38 genes were then analyzed considering miRNAs altered in aggressive PC versus normal tissues, by looking to those genes which are possible targets of PC-altered miRNAs (III approach). With this approach, we reduced the number of PC interesting genes from 38 to 21 (Table 2). Among the 21 genes, several proteins, such as Tp63 transcription factor, Scavenger Receptor Class A Member 3 (*SCARA3*/*CSR1*), and others, demonstrated to have a role in the control of PC cell growth, migration, and metastasis [58,59]. In this group of 21 genes, only three were upregulated (*TRIB1*, *ZDHHC11*, *DPY19L2*). About their regulating miRNA, *hsa-miR-10a* has been already proposed as a candidate circulating biomarker for PC patients [60], while, for the other two miRNAs (*hsa-miR-552* and *hsa-miR-323-3p*), no publication is available. Among the miRNAs regulating the group of downregulated genes, *hsa-miR-182* has been already described as a possible, early diagnostic and prognostic biomarker of PC patients [61], as it is able to promote in vitro proliferation and invasion of PC cell lines [62,63]. The same role in invasion and proliferation has been described for *hsa-miR-17* [64] in PC cells. Similarly, also *hsa-miR-141* has been found in high Gleason score PC cells [65] and has been proposed as a circulating PC biomarker [66]. Furthermore, both *hsa-miR-141* and *hsa-miR-182* have a demonstrated a role in androgen receptor pathway control [67].

Among the 21 genes, we then considered those genes found previously in published gene signatures (IV approach) and we focused on the four genes discussed below.

In the V approach, the co-expression network associated with the identified four genes allowed to place these four genes in a more extended network of 19 co-expressed genes, in which the *hsa-miR-153* seems to regulate a significant higher number of target genes (VI approach).

In the following discussion, we synthetically describe the main affected pathways related to each of the signatures found altered in aggressive PC using these last three approaches.

### 3.1. IV Four-Gene Signature

The four-gene signature identified by comparison of our 21 genes with the published gene signatures, contains three downregulated genes, i.e., *CLU*, Kruppel-like factor 5 (*KLF5*), and *EPHA3*, and one upregulated gene, i.e.,Tribbles pseudokinase 1 (*TRIB1*).

The *CLU* gene codifies for two transcript variants of clusterin protein. CLU1 protein is the most abundant and is present in PC, while CLU2 protein is encoded by the longest transcript and is almost absent in PC cells. CLU mRNA encodes for a stress-inducible, secreted apolipoprotein (also called ApoJ), hypermethilated, thus silenced, in PC tissue [68]. CLU was found to regulate apoptosis, cell–cell interactions, protein stability, cell signalling, proliferation and, finally, transformation [69].

In cancer, *CLU* has been shown to be either up- or downregulated, although the data available on the Oncomine web site show that, in most cancer types, *CLU* is downregulated. In eight out of eight studies, *CLU* expression was found inversely proportional to the grade and/or metastatic stage of PC [70].

Recently, it has been demonstrated that *CLU* expression is regulated by epigenetic mechanisms at the promoter level, as demonstrated by the fact that *CLU* transcription is affected by epigenetic drugs, such as histone deacetylases inhibitors, or DNA methyltransferase inhibitor [71]. Oligonucleotides for *CLU* modulation have been proposed as a potential therapeutic approach for the delayed progression of PC [66], especially for chemotherapy-resistant forms of PC [72,73].

The second gene, *KLF5*, belongs to a family of zinc finger proteins whit transcriptional control activity. The encoded protein promotes cell proliferation, in particular in the absence of TGF-β [74]. Moreover, it seems to control the differentiation of prostatic cells, in particular by modulating the epithelial–mesenchymal transition (EMT) process [75]. *KLF5* loss also promotes the angiogenesis of new microvessels, by upregulation of hypoxia-inducible factor 1-alpha (HIF1α) and its targets, the pro-angiogenic factors vascular endothelial growth factor (*VEGF*) and platelet-derived growth factor (*PDGF*) [76,77].

*EPHA3* gene encodes an ephrin receptor member, with protein tyrosine kinase properties. In PC, it enhances the proliferation and survival of PC cells, both in cellular models, mouse models, and clinical specimens [78]. In particular, the authors found a positive correlation between the levels of *EPHA3* and the Gleason score of PC specimens [78].

*TRIB1*, a member of the Trib family of serine/threonine kinase-like proteins, supports prostate tumorigenesis, and, in a xenograft model of human PC, *TRIB1* depletion strongly inhibited tumor formation [79]. *TRIB1* is an essential factor for PC cell growth and survival and it is involved in the regulation of nuclear factor κB (NF-κB) and mitogen-activated protein (MAP) kinases [79].

### 3.2. V 19-Gene Signature 

The network of 19 co-expressed genes is mainly composed of proteins belonging to three main pathways of the cell life:

1. Phosphatidylinositol-3-kinases (PI3K/AKT) pathway, which include, for example, the ephrin family members (EFNA1, A3, A4, A5, EPHA3, …), the proto-oncogene *LYN*, and tenascin C (TNC);
2. Cell cycle control and proliferation pathways, which include, for example, the Myb-related protein B (MYBL2) and the retinoic acid receptor beta (RARB);
3. Protein Transcription and half-life pathways, which include, for instance, the enhancer binding proteins CEBPB and D, the ribosome binding protein 1 (RRBP1), the ubiquitin protein ligase FBXW7, and KLF5.

For some of these proteins, a role in PC development has been already described. In fact, apart from CLU, also CEBPB and D seem to play a role in PC proliferation, as interleukin-6 (IL-6) treatment increases the expression of CEBP-D family member, inducing IL-6/STAT3-dependent growth arrest on prostate cancer cells in vitro [80].

### 3.3. VI miRNA Signature: hsa-miR-153

Four of the 19 genes (*EPHA3*, *KLF5*, *EFNA5*, and *EFNA3*) of the described gene co-expression signature are possible targets of *hsa-miR-153*. In lung cancer, this miRNA has a role in inhibiting migration and invasion by controlling AKT pathway which promotes tumor growth [81,82]. Its role of tumor suppressor miRNA has been suggested also for glioblastoma [83] and for breast cancer [84]. On the contrary, in PC tissue samples, this miRNA has been found upregulated, and it has been suggested that its upregulation induces cell proliferation, controlling PTEN tumor suppressor mRNA, increasing cyclin D1 expression, and decreasing p21(Cip1) mRNA [85].

Although a biological experimental validation of miRNA silencing and its relation to the expression of the four-gene signature is lacking in this work, our in-silico results show that *hsa-miR-153* may represent a single miRNA-based signature potentially suitable to be used in clinical non-invasive tests and at limited costs for a diagnostic purpose and may thus open new therapeutic approaches in PC.

Analysing the literature, *miR-153* has been already isolated in circulating biofluids (i.e., plasma and whole blood) in several pathological conditions, although not related to PC [20,21,22].

Compared to other miRNAs signatures that include multiple miRNAs [86,87], a signature with only one miRNA could be more stable. A classification based on a high number of signatures can increase the over-fitting of the classification generating high accuracy, but often it is not reproducible [88]. Furthermore, high accuracy in signatures with multiple miRNAs can be due to the contribution of few miRNAs that offset the worse performance of other miRNAs [88].

## 4. Materials and Methods

### 4.1. Gene and miRNA Expression Analysis

From the PC dataset of the Cancer Genome Atlas (TCGA) database, we considered the gene and miRNA expression levels of 344 PC samples and 52 normal samples (NS). More specifically: (1) The expression level of 20531 genes obtained with IlluminaHiSeq RNASeqV2 and (2) The expression levels of 1046 miRNAs identified with Illumina Genome Analyzer miRNA Sequencing. We used 344 primary solid PC samples with Gleason Score equal to or greater than 7 and all 52 matched normal samples with respect to mRNA and miRNA.

Gleason score > 7 is associated with a worse prognosis [89,90].

The clinicopathological characteristics of PC patients and controls are reported in Table 5.

We applied the Differential Expression Analysis on those mRNA transcripts and miRNA which had a mean across the samples higher than the 0.25 * quantile mean [91].

To determine whether a gene or a miRNA was expressed in a differential way, we applied a test of hypothesis, and the fold-change between the two starting conditions in aggressive PC and normal conditions was calculated. We employed the edgeR package from Bioconductor that uses the quantile-adjusted conditional maximum likelihood (qCML) method for experiments with a single factor to determine genes and miRNAs differentially expressed [92]. The *p*-values generated from the analyses sorted in ascending order, were corrected using the Benjamini–Hochberg procedure for multiple testing correction [93]. Differentially expressed genes (DEGs) or differentially expressed miRNA between high-Gleason PC and N samples were considered significant if abs(log fold change) (FC) >1 and false discovery rate (FDR) < 0.01. To avoid unbalanced samples, we performed a series of resampling in order to have, for each resampling, an equal number of samples for each class. Finally, we considered DEGs and miRNAs for all the obtained final samples. In case of TCGA data, resampling was carried out seven times.

### 4.2. Analysis of miRNA Targets

miRWalk [94] was used to identify mRNA targets of each miRNA found in the differential expression analysis. We considered mRNA as targets of deregulated miRNAs if they were found in at least five databases between DIANA-mT, miRanda, miRDB, miRWalk, RNAhybrid, PICTAR4, PICTAR5, PITA, RNA22, and TargetScan.

### 4.3. Copy Number Alterations Analysis 

We applied GISTIC [95] to identify regions of the genome that were amplified or deleted. We used Human Hg19 as a reference file including cytoband and gene location information. Thresholds were setting according to GISTIC parameters [95]: regions with a copy number gain above 0.1 were considered amplifications, regions with a copy number loss below 0.1 were considered deletions, segments that contained fewer than four markers were joined to the neighboring segment closest in copy number, regions with q-values below 0.25 were considered significant.

### 4.4. Combination of Gene Expression and Copy Number Alteration

In this phase, the identification of differentially expressed genes with CNAs (gains/losses) was achieved. In particular, by considering the results of the gene expression analysis (i.e., up- and downregulated genes) and of the CNA analysis (i.e., amplified and deleted genes), we selected upregulated genes with copy number gains in PC patients (by selecting genes common to the set of upregulated and the set of amplified genes), and downregulated genes with copy number losses in PC patients (by selecting genes common to the set of downregulated and the set of deleted genes).

### 4.5. Combination of Gene Expression, CNA, and miRNAs

We assumed that, if a miRNA is up-regulated in cancer, it downregulates a gene that can operate as a tumor suppressor or a transcriptional repressor of an oncogene. Similarly, if a miRNA is downregulated in cancer, its target gene is upregulated, which can be an oncogene or a transcriptional repressor of an oncosuppressor. We analyzed the target genes of up- and downregulated miRNAs from PC patients. These target genes were compared with upregulated and amplified genes and downregulated and deleted genes, respectively. We then chose common genes to the set of: (i) downregulated and deleted genes, with their upregulated miRNAs and (ii) upregulated and amplified genes, with their downregulated miRNAs. We defined these genes as core genes.

### 4.6. Prostate Cancer Signatures

Core genes were compared with those identified by Pubmed research. We retrieved the results of Pubmed Search based on “Prostate Gene Signature” and the names of genes identified from the combination of gene expression, CNA, and miRNAs.

The purpose of this comparison was the assessment of a gene signature consisting of genes which were potentially shared with the abovementioned signatures.

### 4.7. Co-Expressed Network

In order to find genes with similar expression, we investigated the role of a gene co-expression network for the genes found in the last approach.

To find a co-expression network, we used GeneMania, a database with validated interactions [96,97]. We used a Fisher’s Exact Test and, if *p*-value < 0.05, we defined a miRNA enriched by target genes in the co-expression network. Fisher’s Exact Test was applied for genes regulated by differentially expressed miRNA and genes in the network.

**Figure 8:**
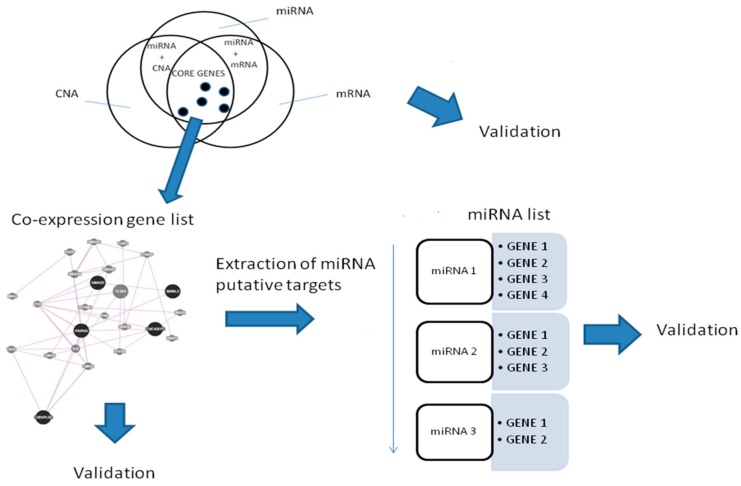
Workflow of the proposed analysis.

A workflow for the procedure is shown in Figure 8.

### 4.8. The Classifier

In order to evaluate the performance of the proposed methodology, we developed a Random Forest (RF) classification model using the R-package [98]. The model was used to classify the considered PC samples versus NS. AUC was estimated by cross-validation method (k-fold cross-validation, k = 10). To avoid unbalanced samples, we did a series of resampling in order to have for each resampling an equal number of samples for each class. Finally, we considered AUC performance as average for all resampling. In the case of TCGA data, resampling was performed seven times.

### 4.9. Evaluation of the Approaches

The performance of the gene signatures in evaluating PC versus NS was validated using candidate biomarkers selected by the different combination approaches: I: The expression levels of the up- or downregulated mRNAs as identified from gene expression analysis; II: The expression levels of the upregulated genes presenting amplification and of the downregulated genes characterized by deletion as found from the combined analysis of gene expression and genome CNA; III: The expression levels of core genes: (i) upregulated and copy number-amplified genes, targets of down-miRNAs; and (ii) downregulated and copy number-deleted genes, targets of up-miRNAs, as identified from the combined analysis of gene expression, genome CNA, and miRNA; IV: The expression levels of genes overlapping with previously established gene signatures; V: The expression levels of genes in the co-expression list based on the core genes; VI: The expression levels of miRNA regulating the network.

The classifier was performed for different gene expression PC datasets. In order to avoid cohort-specific biases, we used PC datasets not employed in any of the above-referenced studies in the process of gene signature identification: 153 PC vs. 49 normal human samples from the GSE79021 dataset. From the miRNA dataset GSE21036: 113 PC patients vs. 28 normal samples.

## 5. Conclusions

The identification of gene and miRNA biomarkers for both the early detection and prognosis of PC is a current challenge. However, currently, there are only few clinical trials with such purposes [99,100,101]. We hypothesized that gene and miRNA signatures of PC could be found by identifying sub-pathways of co-regulated genes that are targeted by specific miRNAs. This co-regulated gene network was built starting from single genes selected by an integrative approach based on three of the most studied modifications in cancer: genes, copy numbers, and miRNAs.

Integrative approaches are based on the principle that the malignant phenotype builds upon multiple molecular phenomena. Thus, the study of different layers of genomic data can better explain different biological mechanisms.

Various layers of genomic data have been identified as DNA, mRNA, miRNA, protein levels, epigenomic features that are associated with tumour aggressiveness, response to therapy, and patient outcome. Moreover, single genes belonging to different signatures are poorly shared among signatures even if they show similar prediction ability of outcomes.

By integrating mRNA expression profiles, CNAs, and miRNA expression levels, we identified a gene signature of four genes overlapping with other published gene signatures, able to distinguish, in silico, high Gleason-scored PC versus normal human tissue. This last signature (four genes, i.e., *TRIB1*, *CLU*, *KLF5*, *EPHA3*) considers not only the biological mechanism underpinning multiple signatures, but also a specific network involved in PC oncogenesis. From this network, we further found one miRNA, *hsa-miR-153*, highly connected to the gene network. This new signature, being able to target multiple genes of a network, acts in regulating distinct biological processes, i.e., PI3K/AKT pathway or protein transcription.

In conclusion, the approach used in our work allowed to identify: (1) a gene signature of four co-expressed genes and (2) a signature of miRNA with a strong role in the regulation of the identified gene network able to diagnose PC. In particular, *hsa-miR-153*, once validated in a laboratory assay, could be suitable for translation to a clinical environment, being easy detectable and possibly measurable by non-invasive tests in circulating biofluids.

## Figures and Tables

**Table 1:** Altered genes from the combined analysis of gene expression and copy number alterations in aggressive prostate cancer.

Alteration	Gene	Position
Upregulated and amplified	*AMY2B*	1p21.1
Upregulated and amplified	*CLEC18B*	16q23.1
Upregulated and amplified	*DPY19L2*	12q14.2
Upregulated and amplified	*GUSBP3*	5q13.2
Upregulated and amplified	*LOC157381*	8q24.21
Upregulated and amplified	*LOC391322*	22q11.23
Upregulated and amplified	*LOC728855*	1q21.2
Upregulated and amplified	*MLIP*	6p12.1
Upregulated and amplified	*POU5F1B*	8q24.21
Upregulated and amplified	*PVT1*	8q24.21
Upregulated and amplified	*SYCE1*	10q26.3
Upregulated and amplified	*TARP*	7p14.1
Upregulated and amplified	*TRIB1*	8q24.21
Upregulated and amplified	*ZDHHC11*	5p15.33
Downregulated and deleted	*ADAMTSL3*	15q25.2
Downregulated and deleted	*ADRA1A*	8p21.2
Downregulated and deleted	*CES1P1*	16q12.2
Downregulated and deleted	*CHRFAM7A*	15q13.2
Downregulated and deleted	*CLU*	8p21.2
Downregulated and deleted	*DMBT1*	10q26.13
Downregulated and deleted	*EPHA3*	3p11.1
Downregulated and deleted	*ETS2*	21q22.2
Downregulated and deleted	*EYA1*	8q13.3
Downregulated and deleted	*FCGR3B*	1q23.3
Downregulated and deleted	*FILIP1*	6q14.1
Downregulated and deleted	*FMN2*	1q43
Downregulated and deleted	*GSTM1*	1p13.3
Downregulated and deleted	*HSPA6*	1q23.3
Downregulated and deleted	*HSPA7*	1q23.3
Downregulated and deleted	*KLF5*	13q22.1
Downregulated and deleted	*MPP2*	17q21.31
Downregulated and deleted	*NAGS*	17q21.31
Downregulated and deleted	*PNMA2*	8p21.2
Downregulated and deleted	*SCARA3*	8p21.2
Downregulated and deleted	*SPG20*	13q13.3
Downregulated and deleted	*THSD7B*	2q22.1
Downregulated and deleted	*TP63*	3q28
Downregulated and deleted	*ZNF826P*	19p12

**Table 2:** List of upregulated and amplified, and downregulated and deleted genes with their candidate miRNA target.

Alteration	Gene Name	miRNA
Upregulated and amplified	*TRIB1*	*hsa-miR-10a*
Upregulated and amplified	*ZDHHC11*	*hsa-miR-552*
Upregulated and amplified	*DPY19L2*	*hsa-miR-323-3p*
Downregulated and deleted	*CLU*	*hsa-miR-217*
Downregulated and deleted	*SCARA3*	*hsa-miR-182*
Downregulated and deleted	*TP63*	*hsa-miR-141*, *hsa-miR-217*
Downregulated and deleted	*HSPA6*	*hsa-miR-17*
Downregulated and deleted	*EYA1*	*hsa-miR-103*
Downregulated and deleted	*MPP2*	*hsa-miR-103*
Downregulated and deleted	*FILIP1*	*hsa-miR-129-5p*
Downregulated and deleted	*DMBT1*	*hsa-miR-197*
Downregulated and deleted	*NAGS*	*hsa-miR-506*
Downregulated and deleted	*PNMA2*	*hsa-miR-183, hsa-miR-217*
Downregulated and deleted	*THSD7B*	*hsa-miR-183*
Downregulated and deleted	*FCGR3B*	*hsa-miR-149*
Downregulated and deleted	*KLF5*	*hsa-miR-148a*, *hsa-miR-217*, *hsa-miR-182*, *hsa-miR-141*
Downregulated and deleted	*FMN2*	*hsa-miR-101*
Downregulated and deleted	*ETS2*	*hsa-miR-182*
Downregulated and deleted	*SPG20*	*hsa-miR-17*
Downregulated and deleted	*EPHA3*	*hsa-miR-182*, *hsa-miR-103*, *hsa-miR-197*, *hsa-miR-153*, *hsa-let-7f*, *hsa-miR-506*, *hsa-miR-454*, *hsa-miR-507*, *hsa-miR-196a*, *hsa-miR-489*, *hsa-miR-513a-3p*, *hsa-miR-1283*, *hsa-miR-103*
Downregulated and deleted	*ADAMTSL3*	*hsa-miR-103*, *hsa-miR-101*, *hsa-miR-19a/b*, *hsa-miR-491-3p*, *hsa-miR-19b*, *hsa-miR-129-5p*, *hsa-miR-142-5p*, *hsa-miR-155*, *hsa-miR-15b*, *hsa-miR-507*, *hsa-miR-512-5p*, *hsa-miR-513a-5p/3p*, *hsa-miR-103*

**Table 3:** List of considered gene signatures.

Samples Used to Generate PC Signature	Author	N. Genes	Common Genes with Our Signature
3D spheroid cell culture model	Mashima et al. [43]	1	*TRIB1*
41 patients with no therapy	Rizzi et al. [44]	8	*CLU*
LNCaP cell line and three patient PC	Duhagon et al. [45]	66	*KLF5*
Xenografts and cell line	Özdemir et al. [46]	3	*EPHA3*

**Table 4:** Gene co-expression from GeneMania.

Entity 1	Entity 2	References to Create the Network
*CEBPB*	*CEBPD*	[Arijs-Rutgeerts-2009, Bahr-Bowler-2013, Mallon-McKay-2013, Roth-Zlotnik-2006, Salaverria-Siebert-2011, Wang-Maris-2006, Wu-Garvey-2007]
*EFNA1*	*EFNA4*	[Bild-Nevins-2006 B, Innocenti-Brown-2011]
*EFNA3*	*EFNA4*	[Innocenti-Brown-2011, Salaverria-Siebert-2011]
*EFNA1*	*EFNA3*	[Gysin-McMahon-2012, Innocenti-Brown-2011]
*ALOX12*	*EPHA3*	[Ramaswamy-Golub-2001, Wang-Maris-2006]
*LYN*	*RRBP1*	[Alizadeh-Staudt-2000, Rieger-Chu-2004]
*CEBPB*	*TRIB1*	[Bahr-Bowler-2013, Rieger-Chu-2004]
*CLUL1*	*CLU*	[Mallon-McKay-2013]
*EFNA1*	*CEBPD*	[Gysin-McMahon-2012]
*MYBL2*	*CLUL1*	[Cheok-Evans-2003]
*TNC*	*CLU*	[Ramaswamy-Golub-2001]
*EFNA3*	*EFNA5*	[Roth-Zlotnik-2006]
*CEBPD*	*TRIB1*	[Bahr-Bowler-2013]
*TNC*	*KLF5*	[Perou-Botstein-1999]
*ALOX12*	*CLU*	[Burington-Shaughnessy-2008, Gysin-McMahon-2012]
*EFNA1*	*CLUL1*	[Rieger-Chu-2004]
*EFNA1*	*KLF5*	[Ramaswamy-Golub-2001, Salaverria-Siebert-2011]
*RRBP1*	*EFNA3*	[Arijs-Rutgeerts-2009]
*LYN*	*EFNA5*	[Perou-Botstein-1999]
*EFNA1*	*FBXW7*	[Kang-Willman-2010]
*NUCB2*	*KLF5*	[Wang-Maris-2006]
*EFNA1*	*CEBPB*	[Roth-Zlotnik-2006]
*LYN*	*CLU*	[Perou-Botstein-1999]
*NUCB2*	*RRBP1*	[Rieger-Chu-2004]
*FBXW7*	*EFNA5*	[Roth-Zlotnik-2006]
*LYN*	*CEBPB*	[Ramaswamy-Golub-2001]
*TNC*	*CEBPB*	[Arijs-Rutgeerts-2009]
*EFNA1*	*TNC*	[Ramaswamy-Golub-2001]
*EFNA4*	*CLUL1*	[Gysin-McMahon-2012]
*XRCC6*	*RRBP1*	[Bahr-Bowler-2013]
*EFNA3*	*TRIB1*	[Wang-Maris-2006]

**Table 5:** Clinicopathological characteristics of PC and control samples.

	PC Patients	Controls
**Age**		
43–50	26	5
51–60	135	18
61–70	165	25
>70	18	4
**Gleason Score**		
7	227	
8	43	
9	72	
10	2	

## Supplementary Materials

The following are available online at http://www.mdpi.com/1422-0067/19/3/910/s1.

## Abbreviations

PC	Prostate Cancer
CNA	Copy Number Alteration
NS	Normal Sample
TCGA	The Cancer Genome Atlas
