# Supplementary figures and images for: In-Silico Integration Approach to Identify a Key miRNA Regulating a Gene Network in Aggressive Prostate Cancer
Source: Int J Mol Sci. 2018 Mar 19;19(3):910. doi: 10.3390/ijms19030910 (PMC5877771; doi:10.3390/ijms19030910)

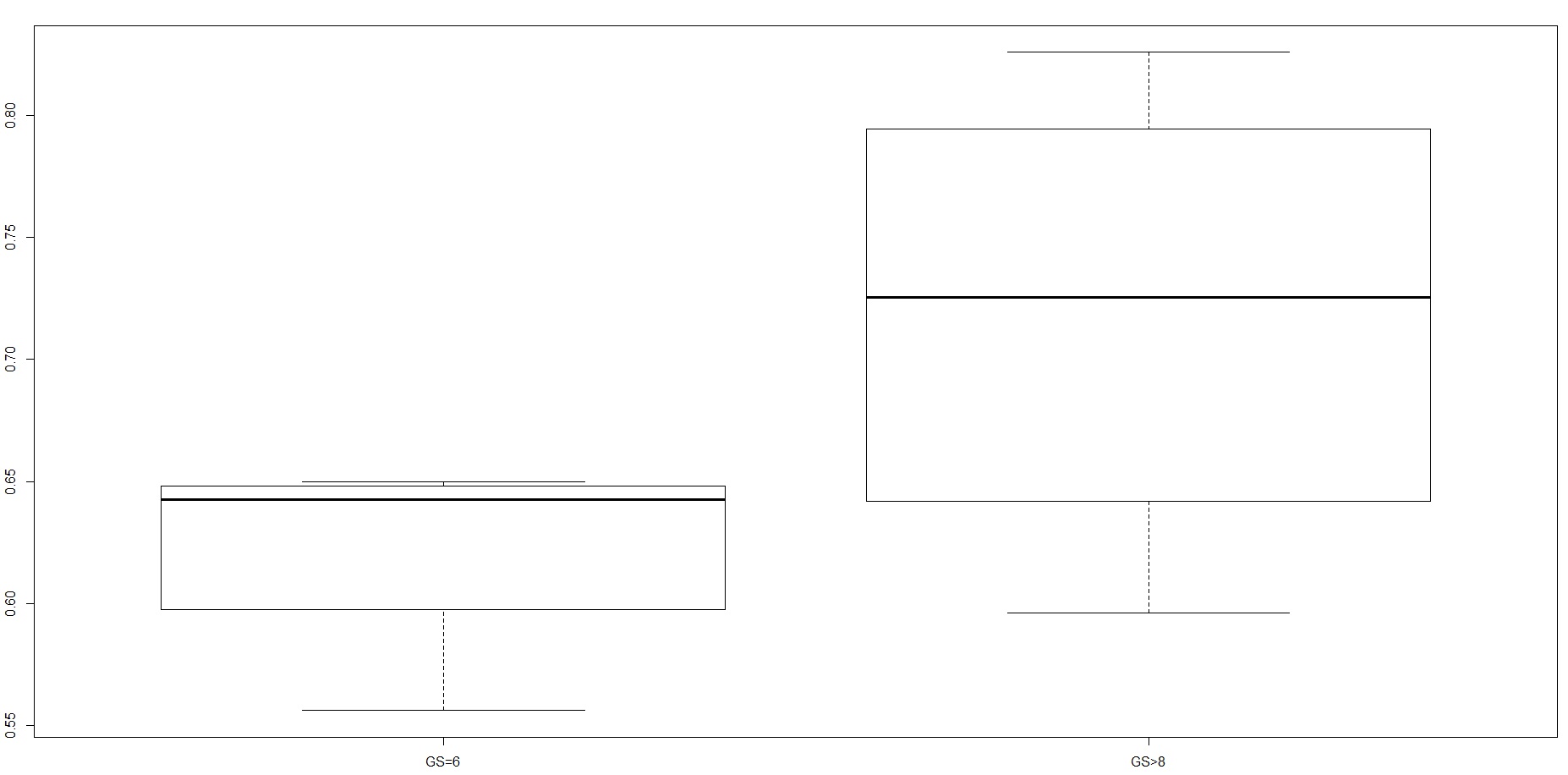

Supplement: Supplementary file 1 [file ijms-19-00910-s001.jpg]
